# Cuprizone-Induced Neurotoxicity in Human Neural Cell Lines Is Mediated by a Reversible Mitochondrial Dysfunction: Relevance for Demyelination Models

**DOI:** 10.3390/brainsci11020272

**Published:** 2021-02-22

**Authors:** Eva Martínez-Pinilla, Núria Rubio-Sardón, Sandra Villar-Conde, Gemma Navarro, Eva del Valle, Jorge Tolivia, Rafael Franco, Ana Navarro

**Affiliations:** 1 Department of Morphology and Cell Biology, Faculty of Medicine, University of Oviedo, 33006 Oviedo, Spain; martinezpinillaeva@gmail.com (E.M.-P.); nuria199510@hotmail.com (N.R.-S.); sandravillarconde@gmail.com (S.V.-C.); valleeva@uniovi.es (E.d.V.); anavarro@uniovi.es (A.N.); 2 Instituto de Neurociencias del Principado de Asturias (INEUROPA), 33003 Oviedo, Spain; 3 Instituto de Investigación Sanitaria del Principado de Asturias (ISPA), 33011 Oviedo, Spain; 4 Department of Biochemistry and Physiology, Faculty of Pharmacy and Food Science, University of Barcelona, 02028 Barcelona, Spain; g.navarro@ub.edu; 5 Centro de Investigación Biomédica en Red Enfermedades Neurodegenerativas (CiberNed), Instituto de Salud Carlos III, 28031 Madrid, Spain; 6 Department of Biochemistry and Molecular Biomedicine, School of Chemistry, University of Barcelona, 08028 Barcelona, Spain

**Keywords:** neurodegenerative diseases, copper chelator, pathophysiology, cell metabolism, glia

## Abstract

Suitable in vivo and in vitro models are instrumental for the development of new drugs aimed at improving symptoms or progression of multiple sclerosis (MS). The cuprizone (CPZ)-induced murine model has gained momentum in recent decades, aiming to address the demyelination component of the disease. This work aims at assessing the differential cytotoxicity of CPZ in cells of different types and from different species: human oligodendroglial (HOG), human neuroblastoma (SH-SY5Y), human glioblastoma (T-98), and mouse microglial (N-9) cell lines. Moreover, the effect of CPZ was investigated in primary rat brain cells. Cell viability was assayed by oxygen rate consumption and by the 3-(4,5-dimethylthiazol-2-yl)-2,5-diphenyltetrazolium bromide-based (MTT) method. Our results demonstrated that CPZ did not cause death in any of the assayed cell models but affected mitochondrial function and aerobic cell respiration, thus compromising cell metabolism in neural cells and neuron-glia co-cultures. In this sense, we found differential vulnerability between glial cells and neurons as is the case of the CPZ-induced mouse model of MS. In addition, our findings demonstrated that reduced viability was spontaneous reverted in a time-dependent manner by treatment discontinuation. This reversible cell-based model may help to further investigate the role of mitochondria in the disease, and study the molecular intricacies underlying the pathophysiology of the MS and other demyelinating diseases.

## 1. Introduction

Multiple sclerosis (MS) is a neurodegenerative, demyelinating, and inflammatory disease of the central nervous system (CNS) that affects more than 2 million people worldwide, with a prevalence two to three times higher in women than in men [1]. This disabling pathology is characterized by the progressive focal loss of oligodendrocytes (OLG) and myelin membranes around axons, which compromises axonal transport and signal transduction, ultimately leading to progressive worsening of symptoms (reviewed in [2,3]). The clinical manifestations appear in multiple fully or partially reversible episodes, and four basic disease courses have been defined and termed as clinically isolated syndrome (CIS), relapsing remitting MS (RRMS), secondary progressive MS (SPMS), and primary progressive MS (PPMS) [4,5]. A myriad of treatments is currently in use in an attempt to alleviate symptoms or slow down MS progression [4,6,7,8,9,10]. Although some are promising in early stages of disease, their long-term effects remain uncertain [4,5,11,12,13,14,15].

The convergence of genetic inheritance, hormonal changes, and environmental factors has long been suspected in determining the risk of developing MS. Despite research efforts in the last years, the mechanisms underlying the MS pathophysiology are still unclear [16]. It seems that neurodegenerative processes driven by inflammation-like events appear at some point in the course of the disease. However, the so-called “inside-out” model of MS pathogenesis is gaining momentum in recent years [17,18,19]. Now, the debate focuses on the exact contribution of each of the mechanisms contributing to axonal damage, which are not mutually exclusive. Obviously, the advance in understanding cellular and molecular pathogenic mechanisms of MS is key to develop effective therapies. In total, three are the main animal models of MS that partially and complementary mimic the different features of human MS: experimental autoimmune encephalomyelitis (EAE), the Theiler’s murine encephalomyelitis virus (TMEV) infection-based model, and the toxin-induced models of demyelination using either cuprizone (CPZ) or lysolecithin [20,21,22]. The autoimmune models are extensively used in the development of new therapies for MS, however, when it comes to further studying myelination processes the CPZ-induced demyelination constitutes the best experimental approach [23,24,25].

CPZ, bis-cyclohexanone oxaldihydrazone, is a cooper chelator that impacts on the activity of Cu^2+^ dependent metalloenzymes such as monoamine oxidase, cytochrome oxidase or Cu^2+^/Zn^2+^-superoxide dismutase. Diverse altered processes, resulting from enzyme inhibition, compromise energy metabolism and increase oxidative stress [25]. In the many studies performed with rodents fed with CPZ, acute OLG degeneration and myelin sheath disruption was followed by spontaneous remyelination after ceasing the CPZ treatment [22,26]. Nevertheless, it has been shown that the exogenous addition of Cu^2+^ does not reduce the CPZ-induced toxicity thus indicating that it is not only acting as ion chelator and other mechanisms may be sought [27,28]. A major question regarding the use of CPZ is the molecular mechanism of action by which the toxic induces OLG death and causes the pathology. To provide reliable answers requires the development of simpler models, and for this purpose cell lines are instrumental.

This work evaluates the cytotoxicity of CPZ in cell lines and highlights mitochondria as a key factor in the neurodegeneration associated with MS. The study includes both cell lines and primary cells.

## 2. Materials and Methods

### 2.1. Cell Lines

HOG cell line, established from a surgically removed human oligodendroglioma by Dr. A. T. Campagnoni (University of California, UCLA, Berkeley, CA, USA) [29] was kindly provided by Dr. J. A. López-Guerrero (Universidad Autónoma de Madrid, Madrid, Spain) [30]. Cells were grown in Dulbecco’s Modified Eagle Medium (DMEM), low glucose, pyruvate, HEPES (22320-022, Invitrogen, Paisley, Scotland, UK), 100 units/mL penicillin and streptomycin (17-602E, Invitrogen, Paisley, Scotland, UK), and 10% (*v*/*v*) heat inactivated fetal bovine serum (FBS) (10270-106, Invitrogen, Paisley, Scotland, UK).

Human neuroblastoma SH-SY5Y cell line was obtained from Sigma (94030304, Sigma-Aldrich, St. Louis, MO, USA) and was grown in DMEM supplemented with 2 mM L-glutamine (61965-059, Invitrogen, Paisley, Scotland, UK), 100 units/mL penicillin and streptomycin (17-602E, Invitrogen, Paisley, Scotland, UK), 1% nonessential amino acids (11140-035, Invitrogen, Paisley, Scotland, UK), and 10% (*v*/*v*) heat inactivated FBS (10270-106, Invitrogen, Paisley, Scotland, UK).

Human glioblastoma T-98 cell line (ATCC) was grown in Eagle’s Minimum Essential Medium (EMEM) supplemented with 100 units/mL penicillin and streptomycin (17-602E, Invitrogen, Paisley, Scotland, UK), and 10% (*v*/*v*) heat inactivated FBS (10270-106, Invitrogen, Paisley, Scotland, UK).

Finally, mouse microglial N-9 cells (ATCC) were cultured in Roswell Park Memorial Institute (RPMI) medium containing 100 units/mL penicillin and streptomycin (17-602E, Invitrogen, Paisley, Scotland, UK), and 10% (*v*/*v*) heat inactivated FBS (10270-106, Invitrogen, Paisley, Scotland, UK).

Cells were maintained at 37 °C in a humidified atmosphere of 5% CO_2_ and were passaged when they were 80–90% confluent, i.e., approximately twice a week for no more than 20 passages. The experiments were always done with cells from passages 3–15.

### 2.2. Co-Cultures

HOG and SH-SY5Y cells were co-cultured in a 2:1.5 proportion, respectively, in DMEM supplemented with 2 mM L-glutamine (61965-059, Invitrogen, Paisley, Scotland, UK), 100 units/mL penicillin and streptomycin (17-602E, Invitrogen, Paisley, Scotland, UK), 1% nonessential amino acids (11140-035, Invitrogen, Paisley, Scotland, UK), and 10% (*v*/*v*) heat inactivated FBS (10270-106, Invitrogen, Paisley, Scotland, UK). The mixed culture was grown at 37 °C in a humidified atmosphere of 5% CO_2_ for at least 48 h before the treatments.

### 2.3. Primary Cultures of Neurons

Cortical neurons were prepared from Sprague–Dawley embryos. Neurons were isolated as described in [31] and plated at 40,000 cells/0.32 cm^2^ confluence. Cortical cells were grown for 12 days in Neurobasal medium (NB) supplemented with 2 mM L-glutamine, 100 units/mL penicillin and streptomycin, and 2% (*v*/*v*) B27 supplement (Invitrogen, Paisley, Scotland, UK) in a 6-well plate. Animal handling was conducted at all times in accordance with the European Council Directive 2010/63/UE, as well as in keeping with current Spanish legislation (RD53/2013). The experimental design was reviewed and approved by the Ethical Committee for Animal Testing of the University of Barcelona.

### 2.4. Cell Treatments

Cell treatments were performed using 3.000–5.000 cells per well in 96-well plates for MTT reduction assay (MTT is a water-soluble tetrazolium reagent in REDOX reactions involving mitochondrial components), 40,000 cells per well in 96-well plates for oxygen consumption assay (OCR), and 30,000 cells per well in 6-well chambered for Trypan blue exclusion assay (an azo blue dye that selectively colors death cells).

For experiments, a stock solution of 30 mM CPZ (C9012-25G, Sigma-Aldrich, St. Louis, MO, USA) was prepared freshly. For this, CPZ powder was dissolved in 50% ethanol/medium shaking at 225 rpm at 60 °C for 15–20 min until its complete dissolution. Working solutions were prepared by diluting the stock in the specific medium for each cell type. After 24–48 h of plating (30–40% cell confluence), cellular toxicity was induced by the addition of CPZ in growing concentrations (0.05–1 mM; see corresponding figure legends), for 24, 48, or 72 h. The CPZ concentrations and times of treatments used in these experiments were based on the bibliography [32,33].

### 2.5. Oxygen Consumption Rate (OCR) Assay

Oxygen consumption was monitored in oxygen-sensing microplates (Oxoprobics Biosciences S. L., Madrid, Spain) as described in [34]. The probe is quenched in the presence of oxygen; as oxygen is consumed by cellular respiration, the fluorescence signal increases being directly related to cell metabolism [35]. Briefly, cells were seeded in oxygen-sensing plates and incubated for 24 h (40–50% cell confluence), the different treatments were added to the cells at the indicated final concentrations, and the wells were sealed from ambient oxygen by the addition of 100 μL/well of mineral oil. Plates were placed in a plate reader (Envision, Perkin–Elmer, Waltham, MA, USA) previously equilibrated at 37 °C and monitored using 340/665 nm excitation/emission filters, with a delay time of 70 μs for 24–48 h.

### 2.6. MTT Reduction Assay

Cell viability was studied by 3-(4,5-dimethylthiazol-2-yl)-2,5-diphenyltetrazolium bromide (MTT) reduction assay, a method based on the activity of mitochondrial NADH-dependent oxidoreductases as indicator of the functional state of mitochondria, as described in [34]. After 24–48 h of plating (30–40% cell confluence) and once treatments were completed, 10 μL of MTT (5 mg/mL in phosphate buffered saline (PBS)) (5655, Sigma-Aldrich, St. Louis, MO, USA) were added to each well. Then, four hours later, 100 μL of lysis solution (20% sodium dodecyl sulfate (SDS); 50% dimethylformamide; pH 4) were added to the culture and incubated overnight at 37 °C. Absorbance at 570 nm was measured using a Multiskan EX Microplate Reader (ThermoFisher Scientific, Waltham, MA, USA). Values from blank dishes, containing only medium, were subtracted from the values of the samples. Cell viability, absorbance at 570 nm, was expressed as the percentage of the controls.

### 2.7. Trypan Blue Exclusion Assay

The trypan blue exclusion test was used to determine the number of dead cells present in a cell suspension by an azo dye that only enters cells with altered membranes. After 24–48 h of plating (30–40% cell confluence) and once treatments were completed, cells were harvested by trypsinization, washed in ice-cold PBS, collected by centrifugation at 200× *g* for 5 min, and examined by adding an equivalent volume of a 0.4% trypan blue solution. The number of live cells (unstained) and dead cells (blue) was counted in a Neubauer chamber (Laboroptik, Arganda del Rey, Madrid, Spain) under light microscopy, and the percentage of viable cells was calculated as described in [36]. In total, four samples were included in each experimental group and each sample was counted at least three times.

### 2.8. Microscopy Observation

Cell cultures were observed using an inverted and phase contrast Nikon Eclipse TS 100 microscope (Nikon, Minato, Tokyo, Japan) and images were recorded by a digital Nikon DS-Fi1 camera (Nikon, Minato, Tokyo, Japan).

### 2.9. Data Analysis

Data result from at least five independent experiments (3–6 replicates). The data in the graphs are presented as the mean ± S.E.M. Statistical analysis was performed with SPSS^®^ 18.0 software (IBM, Armonk, NY, USA). The test of Kolmogorov–Smirnov with the correction of Lilliefors was used to evaluate the fit of the data to a normal distribution and the test of Levene to evaluate the homogeneity of variance. Significance was analyzed by one- or two-way ANOVA test followed by *post-hoc* Tukey’s test for multiple comparisons as described in [34]. Significant differences were considered when *p* < 0.05.

## 3. Results

### 3.1. Cuprizone Decreases Cell Growth but Does Not Cause Death in HOG Cells

**Figure 1:**
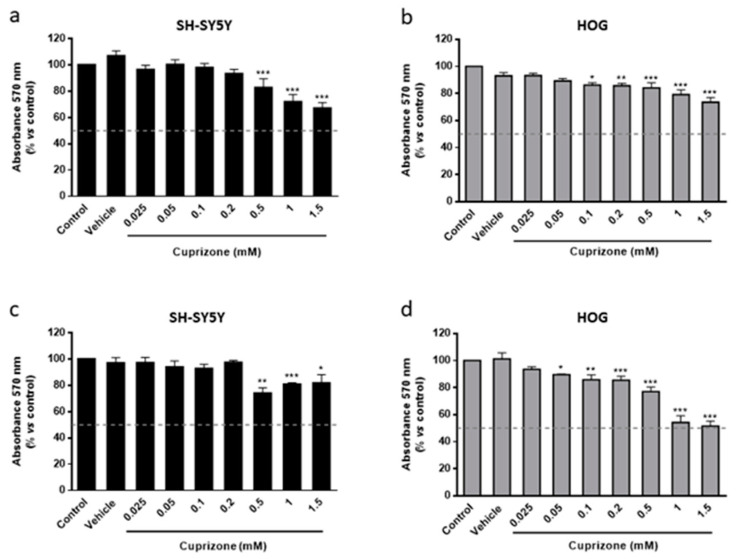
MTT reduction assay in SH-SY5Y (**a**,**c**) and HOG (**b**,**d**) cells treated with increasing concentrations of CPZ (0.025–1.5 mM) for 24 (**a**,**b**) and 48 h (**c**,**d**). Cell damage is represented as the percentage of MTT reduction *versus* control. Data are the mean ± S.E.M of five independent experiments. Significant differences were analyzed by a one-way ANOVA followed by *post-hoc* Tukey’s test. * *p* < 0.05, ** *p* < 0.01, *** *p* < 0.001 compared to control.

We first investigated the cytotoxic effect of CPZ in the human oligodendroglioma HOG cell line using human neuroblastoma SH-SY5Y cells as control. Cell viability was approached by the MTT reduction assay, which is mainly based on reduced activity of mitochondrial enzymes and electron carriers, i.e., is not, as often considered, a marker for cell death but of altered energy production. We will use the term cell viability as synonym of cell having shortage of energy production that if sustained would lead to death. As shown in Figure 1a–d, CPZ induced a significant loss of signal which was concentration-dependent manner in both SH-SY5Y and HOG cells and at either 24 or 48 h of treatment. Data analysis revealed a statistically significant decrease of about 65–70% in cell viability upon 24 h of treatment with 1 mM CPZ. In cells treated for 48 h the extent of toxicity was similar. Importantly, the two cell lines were differentially affected by CPZ, being the OLGs more sensitive to lower concentrations of the toxic than neurons. In fact, HOG cells were significantly affected by 0.1–0.5 mM doses of CPZ, while SH-SY5Y cells were more resistant and higher concentrations (0.5–1.5 mM) were needed to achieve similar effects (Figure 1).

**Figure 2:**
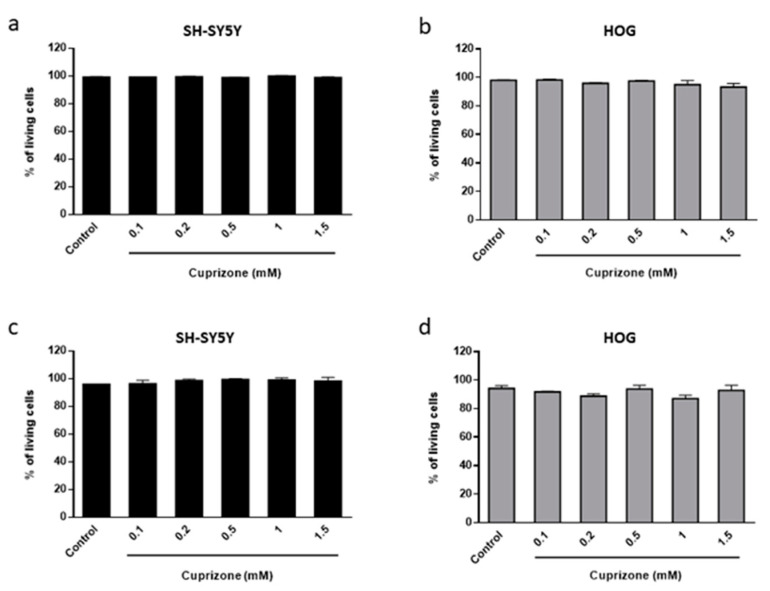
The number of SH-SY5Y (**a**,**c**) and HOG (**b**,**d**) living cells was calculated after 24 (**a**,**b**) and 48 h (**c**,**d**) of culture with increasing concentrations of CPZ (0.1–1.5 mM). Cell viability is represented as the percentage of living cells *versus* control. Data are the mean ± S.E.M of three independent experiments. Each experimental group was run in triplicate, and each plate was counted at least three times.

To assess whether CPZ-induced decrease in cell viability correlates with an increase in cell death, a trypan blue exclusion test was performed. We found that CPZ, at all tested concentrations (0.1–1.5 mM), did not cause death in either SH-SY5Y or HOG cell lines after 24 and 48 h of treatment (Figure 2a–d).

### 3.2. The Toxic Effect on HOG and SH-SY5Y Cells Is Both Mediated by Mitochondria and Reverted Once the CPZ Treatment Is Interrupted

**Figure 3:**
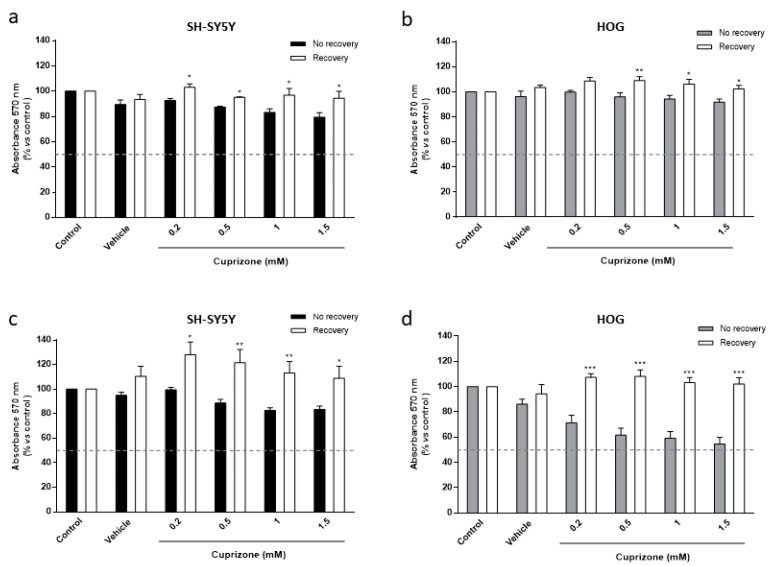
MTT reduction assay in SH-SY5Y (**a**,**c**) and HOG (**b**,**d**) cells treated with increasing concentrations of CPZ (0.2–1.5 mM) for 24 h followed by 24 (**a**,**b**) or 48 h (**c**,**d**) without the treatment (recovery period). Cell damage is represented as the percentage of MTT reduction *versus* control. Data are the mean ± S.E.M of five independent experiments. Significant differences were analyzed by a two-way ANOVA followed by *post-hoc* Tukey’s test. * *p* < 0.05, ** *p* < 0.01, *** *p* < 0.001 compared to no recovery.

As indicated in the introduction section, CPZ-administered mice show a spontaneous remyelination when the treatment is stopped. To better understand the molecular mechanisms underlying this fact, HOG and SH-SY5Y cells were analyzed after a first treatment with CPZ (0.2–1.5 mM) for 24 h followed by growth in fresh medium (recovery period). Results demonstrated the reversion of the toxic effect (60–65%) in both cell lines (Figure 3). It should be noted that oligodendroglioma cells which are more sensitive to CPZ (see above) showed better recovery at longer (48 h) times (Figure 3c,d). Our findings suggest that CPZ affects cell metabolism but that cells recover once the treatment is discontinued.

### 3.3. Cuprizone Alters Mitochondrial Function but Does Not Cause Death in SH-SY5Y/HOG Co-Cultures

One of the major limitations of the in vitro MS models is that culture of a single cell type does not reflect the microenvironment of the CNS. Accordingly, we analyzed the cytotoxic effect of CPZ in neuron-glia in vitro model, namely a SH-SY5Y/HOG cell co-culture.

**Figure 4:**
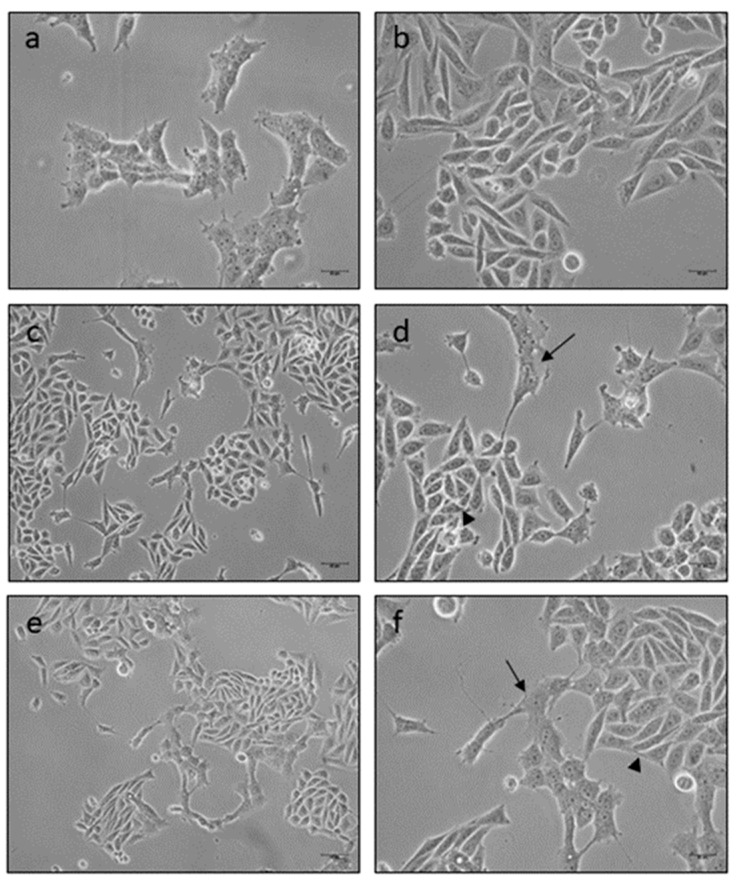
Representative phase contrast inverted microscopy images showing SH-SY5Y (arrowheads) and HOG cells (arrows) grown in a direct physical contact after 48 (**c**,**d**) or 72 h (**e**,**f**) of mixed culture. Cell processes were longer than those in the HOG (**a**) and SH-SY5Y (**b**) cells grown separately during 72 h. (**a**,**b**,**d**,**f**) Scale bars 10 μm (20×); (**c**,**e**) Scale bars 10 μm (10×).

First of all, suitable co-culture conditions were determined in terms of optimal growth medium and cell type proportion (see Material and Methods section). Apart from their morphological identification, we tested by immunofluorescence the presence and proportion of both cell types in the co-cultures, specifically using the anti-NeuN antibody for SH-SY5Y and the anti-B crystallin antibody for HOG cells (data not shown). In these selected conditions, and 24 h after seeding, both cell types were adherent and showed small extended processes. At 48 h of co-culture the cells exhibited conical or fusiform bodies and longer processes (Figure 4c,d). Interestingly, we observed that SH-SY5Y and HOG cells grew in direct physical contact in a 1:1 ratio, approximately. In fact, cells began to establish contacts between them through their extensions at 48 and 72 h of culture (Figure 4c–f). Moreover, HOG cell processes were longer than those in the monocultures (Figure 4a,b,d,f).

**Figure 5:**
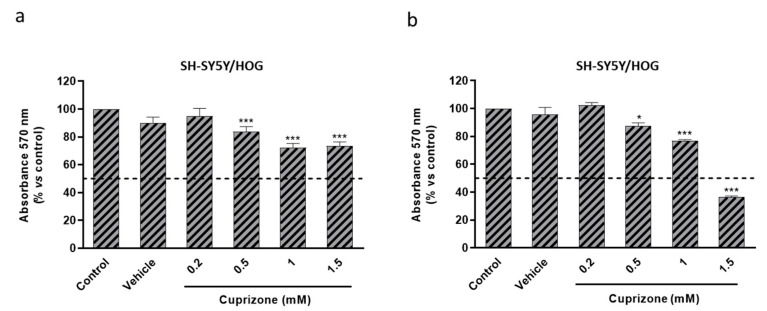
MTT reduction assay in SH-SY5Y/HOG co-cultures treated with increasing concentrations of CPZ (0.2–1.5 mM) for 24 (**a**) and 48 h (**b**). Cell damage is represented as the percentage of MTT reduction *versus* control. Data are the mean ± S.E.M of five independent experiments. Significant differences were analyzed by a one-way ANOVA followed by *post-hoc* Tukey’s test. * *p* < 0.05, *** *p* < 0.001 compared to control.

As it might be expected, CPZ altered the viability of co-cultures in a concentration-dependent fashion. Effects were evident at both 24 and 48 h of treatment. Data analysis revealed a significant decrease (65–70%) in MTT reduction upon 24 h of treatment with 1 mM CPZ (Figure 5a). In cells treated for 48 h the extent of toxicity was similar (Figure 5b).

**Figure 6:**
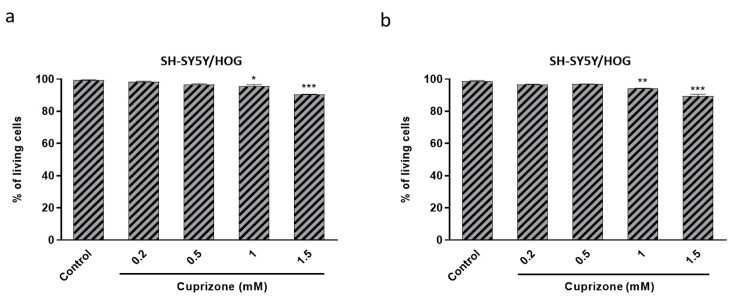
The number of SH-SY5Y and HOG living cells in the co-culture was calculated after treatment with increasing concentrations of CPZ (0.2–1.5 mM) for 24 (**a**) and 48 h (**b**). Cell viability is represented as the percentage of living cells *versus* control. Data are the mean ± S.E.M of three independent experiments. Each experimental group was run in triplicate, and each plate was counted at least three times. Significant differences were analyzed by a one-way ANOVA followed by *post-hoc* Tukey’s test. * *p* < 0.05, ** *p* < 0.01, *** *p* < 0.001 compared to control.

Finally, we tested whether CPZ-induced decrease in cell viability correlated with increase in cell death. The trypan blue exclusion test showed that CPZ caused negligible (5–10%) cell death in SH-SY5Y/HOG co-cultures even at high concentrations (1–1.5 mM) and irrespective of treatment time (24 or 48 h) (Figure 6).

### 3.4. The Loss of Cell Viability Caused by Cuprizone in SH-SY5Y/HOG Co-Cultures Is Reverted Once the Treatment Is Suspended

**Figure 7:**
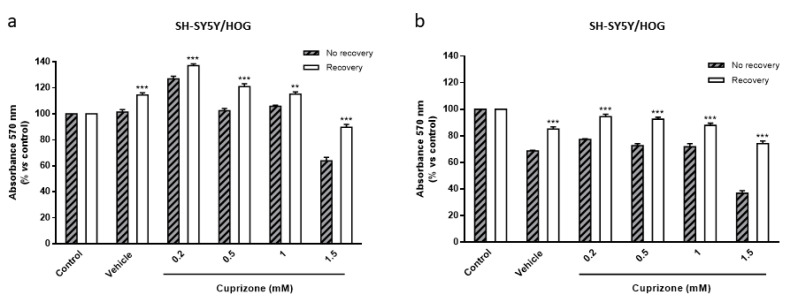
MTT reduction assay in SH-SY5Y/HOG co-cultures treated with increasing concentrations of CPZ (0.2–1.5 mM) for 24 h followed by 24 (**a**) or 48 h (**b**) without the treatment (recovery period). Cell damage is represented as the percentage of MTT reduction *versus* control. Data are the mean ± S.E.M of five independent experiments. Significant differences were analyzed by a two-way ANOVA followed by *post-hoc* Tukey’s test. ** *p* < 0.01, *** *p* < 0.001 compared to no recovery.

The next step was to assess the possibility that the loss of cell viability caused by CPZ could be reverted in SH-SY5Y/HOG co-cultures, as we found for individual cells. Again, we observed a significant increase in cell viability in co-cultures treated with 0.2–1.5 mM CPZ for 24 h following by a recovery period (Figure 7). Moreover, the effect was time-dependent since co-cultures showed greater improvements in viability at 48 than at 24 h of recuperation (Figure 7). These results are consistent with CPZ reversible actions on cell metabolism also detectable in neuron-glia co-cultures.

### 3.5. Oxygen Consumption by the Electron Transport Chain Is Reduced upon CPZ Treatment

**Figure 8:**
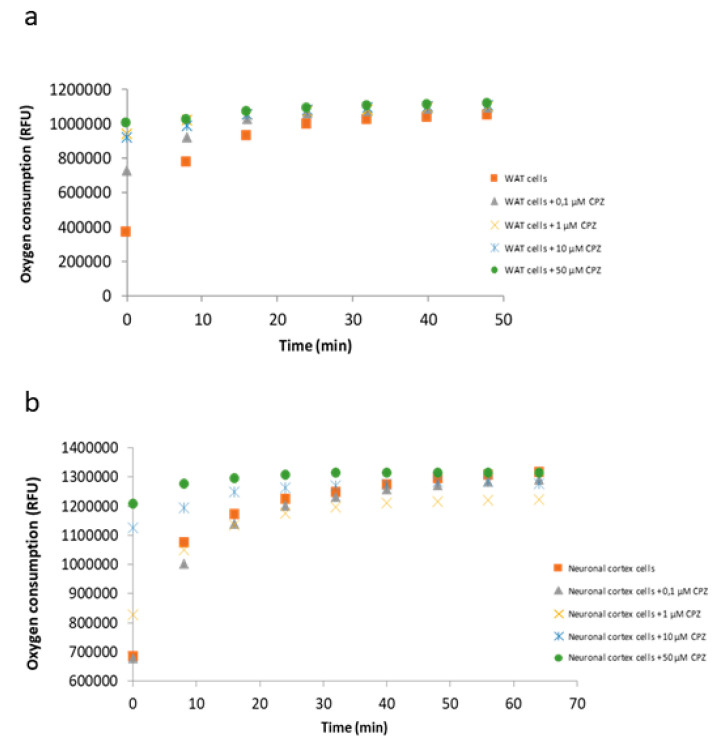
OCR assay in white adipose tissue (WAT) (**a**) and cortical neurons (**b**) incubated with increasing concentrations of CPZ (0.1–50 µM) for 24 h. Oxygen consumption was monitored in real-time using 96-well plate oxygen-sensing plates. Data are the mean ± S.E.M of five independent experiments. RFU: relative fluorescence units.

To better understand the apparently reversible CPZ effect, we tested the oxygen consumption rate (OCR) in primary cultures of neurons from rat neuronal cortex. In our hands, OCR is instrumental to measure the activity of primary cells from white adipose tissue (WAT) and, therefore, we used them as a control (Figure 8). The decrease in OCR induced by CPZ was dose-dependent. Notice that oxygen consumption is a kinetic measure that correlates with the slope of the curves. As shown in Figure 8a,b, the slope was negligible when either primary WAT or cortical cells were treated with 50 µM CPZ. Then, a decrease in slope, in OCR and, consequently, in cell respiration, was detected by the administration of CPZ at concentrations higher than 10 µM (Figure 8). Surprisingly, those concentrations of CPZ (10 or 50 µM) that did not cause significant reductions in cell viability were able to significantly reduce oxygen consumption.

**Figure 9:**
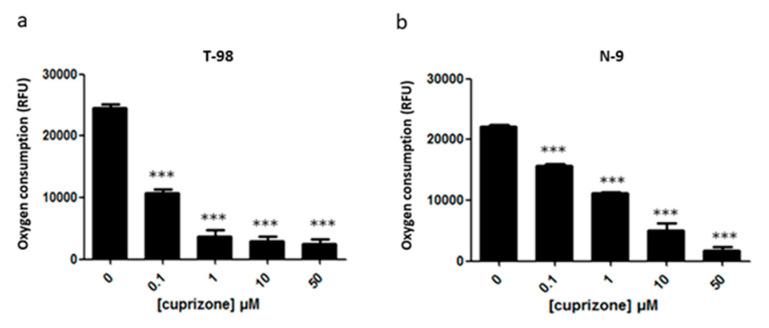
OCR assay in T-98 (**a**) and N-9 cells (**b**) incubated with increasing concentrations of CPZ (0.1–50 µM) for 24 h. Data are the mean ± S.E.M of five independent experiments. Significant differences were analyzed by a one-way ANOVA followed by *post-hoc* Tukey’s test. *** *p* < 0.001 compared to control. RFU: relative fluorescence units.

**Figure 10:**
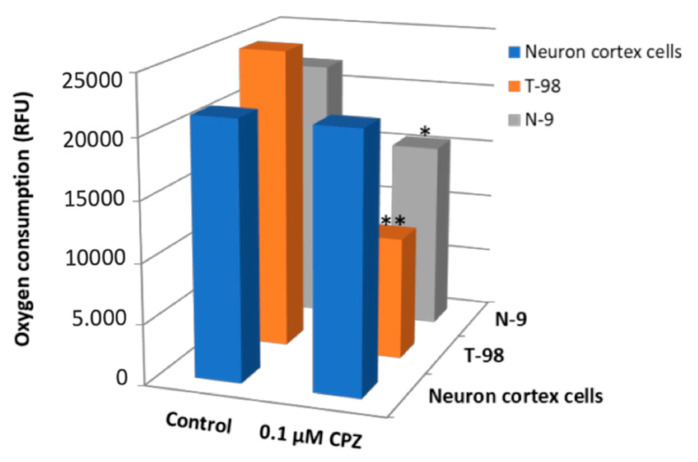
Oxygen consumption in T-98, N-9 and neuronal cortex cells treated with 0.1 µM of CPZ for 24 h. Data are the mean ± S.E.M of five independent experiments. Significant differences were analyzed by a Student’s t-test. * *p* < 0.05, ** *p* < 0.01 glial compared to neuronal cells. RFU: relative fluorescence units.

These results show that energy metabolism is affected by CPZ in fairly different cell types (and not only in neural cells). Then, we aimed at comparing OCR data in primary cultures of neurons with those of glial origin, one from human origin (T-98) with supposedly mild metabolic activity and another from a rodent glioblastoma (N-9) with supposedly high metabolic activity. Our findings show that CPZ caused a concentration-dependent decrease in oxygen consumption in both cell lines (Figure 9). The decline in OCR in T-98 was higher than in the N-9 cells suggesting differential susceptibility to the toxic drug (Figure 9a). Additionally noticeable is the reduction in respiration rate achieved in the two glial cell lines by very low concentrations of CPZ. Significant reductions were already noticed at concentrations as low as 0.1 µM that are ineffective in the case of rat cortical neurons (Figure 10).

## 4. Discussion

The use of in vivo and in vitro models is of pivotal relevance in the study of a complex disease such as MS, characterized by (i) multifactorial etiology, (ii) inter-individual variability of clinical manifestations, and (iii) lengthy time-course. In translational research, these features and the fact that patient samples can only be obtained *post-mortem,* pose significant limitations [11,37]. Nowadays, promoting an early and strong regeneration and remyelination in the CNS appears as the most promising approach in MS treatment, and the CPZ mouse model of demyelination seems to be very appropriate [20,21,22]. Despite its advantages, further work is needed to properly ascertain the intracellular pathways that lead to neuron damage and OLG apoptotic death. Therefore, it is required a simpler model in which molecular mechanism of MS can be better addressed [20,38]. In this sense, we here propose a cell model based on in vitro CPZ toxicity that has already been validated in our laboratory as an effective model to test potential protective molecules that could be key in the development of interventions to afford MS-related neuroprotection [39].

As mentioned, it was difficult to develop MS models in rodents but the CPZ-based mice models are of reference now. The dosage necessary to induce demyelination in rodents is strain- and age-dependent. At an appropriate dosage of CPZ the C57BL⁄ 6 genetic background leads to (mice) models with CNS lesions that are similar to those found in patients [40]. CPZ as copper ion chelator affects mitochondria and, therefore, we wanted to assess the effects in different cell lines. Our results show that CPZ alters mitochondrial function; the compound is mainly toxic for glial cells but it also affects neurons. In fact, the compound impacts on the functional state of mitochondria and aerobic cell respiration, thus compromising cell metabolism. There are very few reports that analyze the effect of CPZ in cellular models. Benetti et al., (2011) observed that treatment with 200 µM of CPZ during 3 weeks did not cause loss of cell viability in SH-SY5Y and GN11 neurons [41]. A later study revealed that the proliferative and survival rates of SH-SY5Y, microglia, astrocytes, and OLG precursor cells were not affected by short-term treatment with different concentrations of CPZ [32]. Recently, it has been demonstrated that CPZ leads to decreases in metabolic viability in the oligodendroglial cell line, MO3.13 [42]. On the one hand, these discrepancies in terms of cytotoxicity may be due to the methodology and the concentration or time of exposure to CPZ. On the other hand, the lack of changes in cell viability, in these previous studies, may be consequence of the Resazurin reduction test chosen, commercially known as AlamarBlue^®^, since it is reported that there are serious concerns about its effectiveness to assess viability [43,44,45].

Also noteworthy is that the effect of CPZ does not translate into glial cell death in contrast to the suggestion derived from in vivo experiments [25]. Demyelinated lesions in the brain of CPZ-intoxicated mice are consequence of OLGs death that correlates with microglial and astroglial activation, and with macrophage accumulation in a highly oxidative and inflammatory scenario characterized by elevated levels of cytokines [28,42,46,47]. In this line, our findings suggest that CPZ cell-specific toxicity, by itself, would not be able to induce OLGs death since immune and inflammatory concomitant cell responses would be required [20]. So far, no significant loss of neurons in the CPZ-mouse model has been reported [26], a finding that fits with our results in primary cultures. Interestingly, we also found that glial cells were more sensitive to CPZ than neurons. OCR results confirm that CPZ may affect virtually all cell types and that the sensitivity of each cell may depend on multiple factors; first of all, on the species as indicated by [40] but also on the cell type. In fact, differential susceptibility of neurons and glia against oxidative or mitochondrial damage in the aged or diseased brains has been the subject of intensive research [48,49]. In a very recent study, Luo et al., (2020) demonstrated a differential vulnerability, in terms of mitochondrial dysfunction, of brain cells of C57BL/6 mice subjected to short-term CPZ intoxication; the more efficient REDOX system of neurons makes them more resistant than OLGs to oxidative stress and mitochondrial damage [50]. The results show that CPZ alters energetic metabolism but why OLGs are more sensitive to these alterations is still an open question.

One simple approach to partially mimic cell responses that take place in the neuronal–glia complex networks of the CNS, is the use of mixed cultures [20,51]. Culture of different cell types in direct contact is useful for some applications but are difficult to obtain, especially when using primary or 3D cell cultures [20]. In this work, we developed a SH-SY5Y/HOG cell co-culture to analyze the effects of CPZ treatment in physiological-like conditions of the CNS. Despite its advantages, there are technical challenges that must be taken into account when one uses a co-culture of two cell lines, i.e., different nutritional requirements or growth rate, which complicates measurements of both intra- and interpopulation interactions and makes the results difficult to interpret. Unlike what one might expect, the effect of CPZ in co-cultures was qualitatively similar to that obtained using individual cell clones.

The CPZ-induced murine model of demyelination has led to an exponential advance in testing novel therapies for MS, especially those aimed at addressing the myelin degenerative aspect of the disease [52]. One of the most relevant conclusions is the spontaneous recovery, to our knowledge never attempted using cell lines. Importantly, our findings demonstrated that the loss of neural viability due to the addition of different concentrations of the copper chelator was reverted in a time-dependent manner upon treatment interruption. This result is in line with the reversibility of demyelination after withdrawal of the toxic in mice subjected for 3–6 weeks to a diet containing 0.2% of CPZ [53]. Gudi et al., (2014) argued that remyelination phenomenon in murine models is based on proliferation and differentiation of precursor OLGs [54]. Bearing in mind our data, we can also hypothesize that mature OLGs functionality is compromised during treatment but returns back to normal after the toxic challenge.

Despite that CPZ model has constituted, for more than 50 years, one of the best models for the study of demyelinating diseases such as MS, the exact mechanism of action of CPZ is not completely understood. Nowadays, the main hypothesis is that CPZ could pass through biological membranes and compromise mitochondrial function acting directly on intracellular Cu (II) ion content, affecting Cu^2+^/Zn^2+^-superoxide dismutase or monoamine oxidase function, and diminishing the activity of the electron transport chain [25,54,55]. The consequent decrease in energy production and the increase in oxidative stress lead to mitochondrial damage and, eventually, to cell death, as it has been shown in vivo in hepatocytes and also in OLGs [56,57]. In this sense, Pasquini et al., (2007) demonstrated that CPZ causes the appearance of megamitochondria in OLGs, a large and swollen dysfunctional form of this subcellular organelle. However, more recently, it has been suggested that CPZ could also act in an ion chelator independent fashion [27,42,58]. In this sense, a recent work by Taraboletti and co-workers (2017) indicates that CPZ uptake causes lipid metabolism disturbances that compromise OLGs survival. Additionally relevant, is the proven relationship between CPZ intoxication, oxidative stress and apoptosis in mature OLGs [25,26,59].

In conclusion, our work demonstrates that CPZ does not cause death in any of the assayed cell models, albeit it induces a significant loss of metabolic performance, may be due to the alteration of mitochondrial function, that is spontaneously restored when the toxic is eliminated. Moreover, we find differential vulnerability between glial cells and neurons. Therefore, the reversible cytotoxic model here implemented may help to further investigate the role of mitochondria in MS-associated neurodegeneration. It constitutes, a suitable approximation to study the interconnected molecular mechanisms underlying the pathophysiology of the MS and other demyelinating diseases, often characterized by periods of exacerbation of neurologic symptoms followed by periods of partial or complete recovery (remissions) as RRMS. Nevertheless, further experimental effort is needed to address whether the cytotoxicity is mediated by a direct or indirect effect of CPZ on mitochondrial electron transport chain.

## Data Availability

The data presented in this study are available on request from the corresponding author. The data are not publicly available due to privacy restrictions.

## References

[1] Ray Dorsey E., Elbaz A., Nichols E., Abd-Allah F., Abdelalim A., Adsuar J.C., Ansha M.G., Brayne C., Choi J.Y.J., Collado-Mateo D. (2018). Global, regional, and national burden of Parkinson’s disease, 1990–2016: A systematic analysis for the Global Burden of Disease Study 2016. Lancet Neurol..

[2] Gelfand J.M. (2014). Multiple sclerosis: Diagnosis, differential diagnosis, and clinical presentation. Handbook of Clinical Neurology.

[3] Noseworthy J.H., Lucchinetti C., Rodriguez M., Weinshenker B.G. (2000). Multiple sclerosis. N. Engl. J. Med..

[4] Correale J., Gaitán M.I., Ysrraelit M.C., Fiol M.P. (2017). Progressive multiple sclerosis: From pathogenic mechanisms to treatment. Brain.

[5] Lublin F.D., Reingold S.C., Cohen J.A., Cutter G.R., Sørensen P.S., Thompson A.J., Wolinsky J.S., Balcer L.J., Banwell B., Barkhof F. (2014). Defining the clinical course of multiple sclerosis: The 2013 revisions. Neurology.

[6] Goldberg P., Fleming M.C., Picard E.H. (1986). Multiple sclerosis: Decreased relapse rate through dietary supplementation with calcium, magnesium and vitamin D. Med. Hypotheses.

[7] Kappos L., Bar-Or A., Cree B.A.C., Fox R.J., Giovannoni G., Gold R., Vermersch P., Arnold D.L., Arnould S., Scherz T. (2018). Siponimod versus placebo in secondary progressive multiple sclerosis (EXPAND): A double-blind, randomised, phase 3 study. Lancet.

[8] Leibowitz S.M., Yan J. (2016). NF-κB pathways in the pathogenesis of multiple sclerosis and the therapeutic implications. Front. Mol. Neurosci..

[9] Ontaneda D., Hyland M., Cohen J.A. (2012). Multiple Sclerosis: New Insights in Pathogenesis and Novel Therapeutics. Annu. Rev. Med..

[10] Vandenbark A.A., Culbertson N.E., Bartholomew R.M., Huan J., Agotsch M., LaTocha D., Yadav V., Mass M., Whitham R., Lovera J. (2008). Therapeutic vaccination with a trivalent T-cell receptor (TCR) peptide vaccine restores deficient FoxP3 expression and TCR recognition in subjects with multiple sclerosis. Immunology.

[11] Barkhof F., Hulst H.E., Drulović J., Uitdehaag B.M.J., Matsuda K., Landin R. (2010). Ibudilast in relapsing-remitting multiple sclerosis: A neuroprotectant?. Neurology.

[12] Brown J.W.L., Coles A., Horakova D., Havrdova E., Izquierdo G., Prat A., Girard M., Duquette P., Trojano M., Lugaresi A. (2019). Association of Initial Disease-Modifying Therapy with Later Conversion to Secondary Progressive Multiple Sclerosis. JAMA.

[13] Burton J.M., Kimball S., Vieth R., Bar-Or A., Dosch H.M., Cheung R., Gagne D., D’Souza C., Ursell M., O’Connor P. (2010). A phase I/II dose-escalation trial of vitamin D3 and calcium in multiple sclerosis. Neurology.

[14] Frau J., Coghe G., Lorefice L., Fenu G., Cocco E. (2018). New horizons for multiple sclerosis therapeutics: Milestones in the development of ocrelizumab. Neuropsychiatr. Dis. Treat..

[15] Ontaneda D., Fox R.J., Chataway J. (2015). Clinical trials in progressive multiple sclerosis: Lessons learned and future perspectives. Lancet Neurol..

[16] Reich D.S., Lucchinetti C.F., Calabresi P.A. (2018). Multiple sclerosis. N. Engl. J. Med..

[17] Stys P.K., Zamponi G.W., Van Minnen J., Geurts J.J.G. (2012). Will the real multiple sclerosis please stand up?. Nat. Rev. Neurosci..

[18] Caprariello A.V., Rogers J.A., Morgan M.L., Hoghooghi V., Plemel J.R., Koebel A., Tsutsui S., Dunn J.F., Kotra L.P., Ousman S.S. (2018). Biochemically altered myelin triggers autoimmune demyelination. Proc. Natl. Acad. Sci. USA.

[19] Titus H.E., Chen Y., Podojil J.R., Robinson A.P., Balabanov R., Popko B., Miller S.D. (2020). Pre-clinical and Clinical Implications of “Inside-Out” vs. “Outside-In” Paradigms in Multiple Sclerosis Etiopathogenesis. Front. Cell. Neurosci..

[20] van Der Star B., Vogel D., Kipp M., Puentes F., Baker D., Amor S. (2012). In Vitro and In Vivo Models of Multiple Sclerosis. CNS Neurol. Disord. Drug Targets.

[21] Palumbo S., Pellegrini S. (2017). Experimental In Vivo Models of Multiple Sclerosis: State of the Art. Multiple Sclerosis: Perspectives in Treatment and Pathogenesis.

[22] Procaccini C., De Rosa V., Pucino V., Formisano L., Matarese G. (2015). Animal models of Multiple Sclerosis. Eur. J. Pharmacol..

[23] Denic A., Johnson A.J., Bieber A.J., Warrington A.E., Rodriguez M., Pirko I. (2011). The relevance of animal models in multiple sclerosis research. Pathophysiology.

[24] Nyamoya S., Schweiger F., Kipp M., Hochstrasser T. (2017). Cuprizone as a model of myelin and axonal damage. Drug Discov. Today Dis. Model..

[25] Vega-Riquer J.M., Mendez-Victoriano G., Morales-Luckie R.A., Gonzalez-Perez O. (2019). Five Decades of Cuprizone, an Updated Model to Replicate Demyelinating Diseases. Curr. Neuropharmacol..

[26] Praet J., Guglielmetti C., Berneman Z., Van der Linden A., Ponsaerts P. (2014). Cellular and molecular neuropathology of the cuprizone mouse model: Clinical relevance for multiple sclerosis. Neurosci. Biobehav. Rev..

[27] Carlton W.W. (1969). Spongiform encephalopathy induced in rats and guinea pigs by cuprizone. Exp. Mol. Pathol..

[28] Messori L., Casini A., Gabbiani C., Sorace L., Muniz-Miranda M., Zatta P. (2007). Unravelling the chemical nature of copper cuprizone. Dalt. Trans..

[29] Post G.R., Dawson G. (1992). Characterization of a cell line derived from a human oligodendroglioma. Mol. Chem. Neuropathol..

[30] Bello-Morales R., Crespillo A.J., García B., Dorado L.Á., Martín B., Tabarés E., Krummenacher C., De Castro F., López-Guerrero J.A. (2014). The effect of cellular differentiation on HSV-1 infection of oligodendrocytic cells. PLoS ONE.

[31] Hradsky J., Mikhaylova M., Karpova A., Kreutz M.R., Zuschratter W. (2013). Super-resolution microscopy of the neuronal calcium-binding proteins calneuron-1 and caldendrin. Methods Mol. Biol..

[32] Bénardais K., Kotsiari A., Škuljec J., Koutsoudaki P.N., Gudi V., Singh V., Vulinović F., Skripuletz T., Stangel M. (2013). Cuprizone [bis(cyclohexylidenehydrazide)] is selectively toxic for mature oligodendrocytes. Neurotox. Res..

[33] Cammer W. (1999). The neurotoxicant, cuprizone, retards the differentiation of oligodendrocytes in vitro. J. Neurol. Sci..

[34] Martínez-Pinilla E., Aguinaga D., Navarro G., Rico A.J., Oyarzábal J., Sánchez-Arias J.A., Lanciego J.L., Franco R. (2019). Targeting CB1 and GPR55 Endocannabinoid Receptors as a Potential Neuroprotective Approach for Parkinson’s Disease. Mol. Neurobiol..

[35] Hynes J., Floyd S., Soini A.E., O’Connor R., Papkovskyi D.B. (2003). Fluorescence-based cell viability screening assays using water-soluble oxygen probes. J. Biomol. Screen..

[36] Martínez E., Navarro A., Ordóñez C., Del Valle E., Tolivia J. (2013). Oxidative stress induces apolipoprotein d overexpression in hippocampus during aging and alzheimer’s disease. J. Alzheimer’s Dis..

[37] Palumbo S. (2017). Pathogenesis and Progression of Multiple Sclerosis: The Role of Arachidonic Acid–Mediated Neuroinflammation. Multiple Sclerosis: Perspectives in Treatment and Pathogenesis.

[38] Buntinx M., Vanderlocht J., Hellings N., Vandenabeele F., Lambrichts I., Raus J., Ameloot M., Stinissen P., Steels P. (2003). Characterization of three human oligodendroglial cell lines as a model to study oligodendrocyte injury: Morphology and oligodendrocyte-specific gene expression. J. Neurocytol..

[39] Martínez-Pinilla E., Rubio-Sardón N., Peláez R., García-Álvarez E., Del Valle E., Tolivia J., Larráyoz I.M., Navarro A. (2021). Neuroprotective Effect of Apolipoprotein D in Cuprizone-Induced Cell Line Models: A Potential Therapeutic Approach for Multiple Sclerosis and Demyelinating Diseases. Int. J. Mol. Sci..

[40] Torkildsen Ø., Brunborg L.A., Myhr K.-M., Bø L. (2008). The cuprizone model for demyelination. Acta Neurol. Scand..

[41] Benetti F., Ventura M., Salmini B., Ceola S., Carbonera D., Mammi S., Zitolo A., D’Angelo P., Urso E., Maffia M. (2010). Cuprizone neurotoxicity, copper deficiency and neurodegeneration. Neurotoxicology.

[42] Taraboletti A., Walker T., Avila R., Huang H., Caporoso J., Manandhar E., Leeper T.C., Modarelli D.A., Medicetty S., Shriver L.P. (2017). Cuprizone Intoxication Induces Cell Intrinsic Alterations in Oligodendrocyte Metabolism Independent of Copper Chelation. Biochemistry.

[43] Chen J.L., Steele T.W.J., Stuckey D.C. (2018). Metabolic reduction of resazurin; location within the cell for cytotoxicity assays. Biotechnol. Bioeng..

[44] O’Brien J., Wilson I., Orton T., Pognan F. (2000). Investigation of the Alamar Blue (resazurin) fluorescent dye for the assessment of mammalian cell cytotoxicity. Eur. J. Biochem..

[45] Uzarski J.S., DiVito M.D., Wertheim J.A., Miller W.M. (2017). Essential design considerations for the resazurin reduction assay to noninvasively quantify cell expansion within perfused extracellular matrix scaffolds. Biomaterials.

[46] Buschmann J.P., Berger K., Awad H., Clarner T., Beyer C., Kipp M. (2012). Inflammatory response and chemokine expression in the white matter corpus callosum and gray matter cortex region during cuprizone-induced demyelination. J. Mol. Neurosci..

[47] Liu L., Belkadi A., Darnall L., Hu T., Drescher C., Cotleur A.C., Padovani-Claudio D., He T., Choi K., Lane T.E. (2010). CXCR2-positive neutrophils are essential for cuprizone-induced demyelination: Relevance to multiple sclerosis. Nat. Neurosci..

[48] Fünfschilling U., Supplie L.M., Mahad D., Boretius S., Saab A.S., Edgar J., Brinkmann B.G., Kassmann C.M., Tzvetanova I.D., Möbius W. (2012). Glycolytic oligodendrocytes maintain myelin and long-term axonal integrity. Nature.

[49] Lassmann H., Van Horssen J. (2016). Oxidative stress and its impact on neurons and glia in multiple sclerosis lesions. Biochim. Biophys. Acta Mol. Basis Dis..

[50] Luo M., Deng M., Yu Z., Zhang Y., Xu S., Hu S., Xu H. (2020). Differential Susceptibility and Vulnerability of Brain Cells in C57BL/6 Mouse to Mitochondrial Dysfunction Induced by Short-Term Cuprizone Exposure. Front. Neuroanat..

[51] Hyung S., Yoon Lee B., Park J.C., Kim J., Hur E.M., Francis Suh J.K. (2015). Coculture of Primary Motor Neurons and Schwann Cells as a Model for in Vitro Myelination. Sci. Rep..

[52] Kolahdouzan M., Futhey N.C., Kieran N.W., Healy L.M. (2019). Novel Molecular Leads for the Prevention of Damage and the Promotion of Repair in Neuroimmunological Disease. Front. Immunol..

[53] Hillis J.M., Davies J., Mundim M.V., Al-Dalahmah O., Szele F.G. (2016). Cuprizone demyelination induces a unique inflammatory response in the subventricular zone. J. Neuroinflammation.

[54] Gudi V., Gingele S., Skripuletz T., Stangel M. (2014). Glial response during cuprizone-induced de- and remyelination in the CNS: Lessons learned. Front. Cell. Neurosci..

[55] Pasquini L.A., Calatayud C.A., Bertone Uña A.L., Millet V., Pasquini J.M., Soto E.F. (2007). The neurotoxic effect of cuprizone on oligodendrocytes depends on the presence of pro-inflammatory cytokines secreted by microglia. Neurochem. Res..

[56] Matsushima G.K., Morell P. (2006). The Neurotoxicant, Cuprizone, as a Model to Study Demyelination and Remyelination in the Central Nervous System. Brain Pathol..

[57] Patergnani S., Fossati V., Bonora M., Giorgi C., Marchi S., Missiroli S., Rusielewicz T., Wieckowski M.R., Pinton P. (2017). Mitochondria in Multiple Sclerosis: Molecular Mechanisms of Pathogenesis. International Review of Cell and Molecular Biology.

[58] Moldovan N., Al-Ebraheem A., Lobo L., Park R., Farquharson M.J., Bock N.A. (2015). Altered transition metal homeostasis in the cuprizone model of demyelination. Neurotoxicology.

[59] Hesse A., Wagner M., Held J., Brück W., Salinas-Riester G., Hao Z., Waisman A., Kuhlmann T. (2010). In toxic demyelination oligodendroglial cell death occurs early and is FAS independent. Neurobiol. Dis..

